# Fatal drowning statistics from the Netherlands – an example of an aggregated demographic profile

**DOI:** 10.1186/s12889-022-12620-3

**Published:** 2022-02-17

**Authors:** Joost Bierens, Jan Hoogenboezem

**Affiliations:** 1 Research Group Emergency and Disaster Medicine, Vrije Universiteit Brussels, Laarbeeklaan 103, 1090 Brussels, Belgium; 2 Centraal Bureau voor de Statistiek (Statistics Netherlands), Department of Causes of Death Statistics, Henri Faasdreef 312, 2492 JP Den Haag, the Netherlands

**Keywords:** Demographic, Drowning, Suicide, Transportation, Ethnicity, Trend

## Abstract

**Introduction:**

Incompleteness of fatal drowning statistics is a familiar problem impeding public health measures. Part of the problem may be that only data on accidental drowning are used and not the full potential of accessible data.

**Methods:**

This study combines cause-of-death certificates and public prosecutor’s court documents between 1998 and 2017 to obtain an aggregated profile. Data are also used as a basis for a trend analysis.

**Results:**

The dataset includes 5571 drowned persons (1.69 per 100,000). The highest risk group are persons above the age of 50. Demographic differences are observed between suicide by drowning, accidental drowning, and drowning due to transportation (0.72, 0.64, 0.28 per 100.000) and between native Dutch, and Dutch with western and non-western background (1.46, 1.43, 1.76 per 100.000). Non-residents account for another 12.2%. When comparing the periods 1998–2007 with 2008–2017, the Standard Mortality declines for suicide drowning and accidental drowning among persons with a native Dutch and non-western background. Single regression analysis confirms a decrease of drowning over the full period, breakpoint analysis shows an increase in the incidence of the total number of drowning, suicide by drowning and accidental drowning starting in 2007, 2008 resp. 2012.

**Discussion:**

Compared to the formal number of fatal accidental drowning in the Netherlands (*n* = 1718; incidence 0.52 per 100,000), the study identifies 350% more drowning. Differences in demographic data and the recent increase needs to be explored for public health interventions.

**Supplementary Information:**

The online version contains supplementary material available at 10.1186/s12889-022-12620-3.

## Introduction

The Global Report on Drowning, published in 2014 by the World Health Organisation (WHO), suggests that formal national data underrepresent the full fatal drowning toll by up to 50% in some high-income countries and up to 400–500% in low and middle-income countries [1, 2]. A lack of reliable national data on drowning has been recognised for over the past two decades. Multiple reasons have been identified to explain this [3–7], including the absence in the national data of fatal drowning by suicide, transportation accidents, natural disasters such as floods [8–11], cyclones [12], tsunamis [13, 14] as well as disasters with ferryboats, cruise ships and migrant crafts [14]. Another explanation for the lack of reliable national data is the exclusion of non-inhabitants such as foreign students, workers, tourists and migrants [15–20] in the national statistics and low quality of data registration and collecting systems [10, 21, 22]. The update in 1990 of the International Classification of Diseases (ICD) from ICD-9 to ICD-10 may have further limited the opportunities to obtain representative national and international data on drowning, although the effect is considered small [6, 23, 24].

A complete and reliable picture of the total burden of fatal, and also non-fatal, drowning is needed to monitor current and future trends [20, 22, 25–27], to understand the impact of various prevention programmes [20, 26, 28] and to become timely informed about the acute health effects in the aquatic environment of countries affected by climate change or other reasons of instability [29]. This may also paradoxically become important now that recent statistics show a sudden and unexplained decrease in drowning rates [30].

Studies that provide evidence of the suggested weakness of data on drowning are scarce [2, 23, 31–33]. This study was initiated with the aim of identifying the complete burden of fatal drowning in one European country, including suicides, transportation accidents and non-residents who drowned fatally while in the Netherlands. The characteristics of the various groups were explored. Comparison of the data between 1998 and 2007 and between 2008 and 2017, as well as trend analysis and breakpoint analysis over the period 1998–2017, allowed the identification of trends over the last 20 years. The methodology used, provides a standardised method that may serve as an example to obtain a more complete profile of drowning, and their trends, in countries with a well-functioning cause of death registration.

## Material and methods

### Setting

Statistics Netherlands (CBS) is responsible for collecting, compiling, producing and publishing causes of death statistics [34]. For every deceased person registered in the Personal Records Database (Basis Registratie Personen: BRP) of the Netherlands, an attending medical practitioner or a forensic doctor appointed by the municipality is obliged by law to complete a Medical Certification of Cause-of-Death (MCCD). The MCCD distinguishes between natural and non-natural (also known as external) causes. In non-natural (external) causes of death, the event directly leading to the death is considered the underlying cause of death. The resulting injury is the code of injury. Based on the MCCD, the underlying causes of death and the codes of injury are encoded in accordance with the tenth revision of the International Statistical Classification of Diseases and Related Health Problems (ICD-10) of the World Health Organisation (WHO). When a MDDC is unclear or incomplete, the certifier is contacted.

For each deceased registered in the BRP, a check is performed whether a MDDC has been received by CBS. More than 99% of the records can be linked to the corresponding MDDC and are then coded based on the ICD-10. For a person registered in the BRP who died abroad, there is usually no death certificate and therefore this is almost always recorded as “Other ill-defined and unspecified causes of mortality” (R99, ICD-10).

In addition, for each case of non-natural death, a court document is created by regional Public Prosecutor’s Offices (PPO). Statistics Netherlands (CBS) investigates such documents and the relevant data related to the cause of death are filled out in a standardised questionnaire that includes information, e.g. on the cause of drowning. In order to optimise the quality of cause- of-death statistics on persons included in the BRP, data from MDDC and court files are combined with CBS data. If needed, data are corrected until both documents contain identical and plausible information before becoming finalised. The PPO court also produces documents with the non-natural causes of death of all non-residents and people with no fixed place of residence.

### Definitions

In this study, the formal definitions by the CBS are used.

#### Resident in the Netherlands

A person living in the Netherlands who is registered in the BRP.

#### Resident with a migration background

A person living in the Netherlands who has at least one parent who was born outside the Netherlands. A person with a Western migration background has at least one parent born in another country in Europe (excluding Turkey), North America, Oceania, Indonesia or Japan. A person with a non-Western migration background has at least one parent born in a country in Africa, Latin America or Asia (excluding Indonesia and Japan) or Turkey [35].

#### Non-residents in the Netherlands

A person not living in the Netherlands or living in the Netherlands for less than 4 months; a non-resident is not registered in the BRP. Non-residents include tourists and other foreign visitors in the Netherlands; persons who temporarily work or study in the Netherlands; undocumented immigrants and asylum seekers who stay in the Netherlands.

### Building up the data file

This study includes all persons who died in the period 1998 to 2017 with drowning as the injury code T75.1 (ICD-10), regardless of the underlying cause of death (external cause). In this way, in addition to accidental drowning (W65-W74), also drowning were included by intentional self-harm, such as suicide by drowning (X60-X84), transport accidents (V01-V99) and a remaining group with various causes (A00-Y99). The latter group includes the residual group of drowning: by murder or manslaughter; persons of whom it is unknown whether they either accidentally or intentionally drowned; persons who died more than 30 days after the drowning accident; late effects of drowning; persons who drowned after an epileptic seizure; and who drowned after falling through the ice.

CBS began to use the ICD-10 in 1996. The CBS data of all fatal drowning of residents that occurred in the Netherlands from 1998 until 2017 were included in the data file. For non-residents who drowned in the Netherlands, the data files selected from the PPO documents were added. For the analysis described in this publication, only the data on gender, date of birth, manner of drowning, country of origin and date of death have been used.

### Presentation of the data

Data on deaths by drowning are presented in absolute numbers, incidence per 100,000 of the average population, Standard Mortality (SM) and Standard Mortality Ratio (SMR). The average population is the mean of the population as at 1 January and as at 31 December of the same year. The incidence in a year is the total number of confirmed deaths divided by the average population over the same period per 100,000 population.

In the total number of deaths by drowning in the Netherlands, non-residents are included. There is no data on the complete population of non-residents, since this population varies greatly throughout the year, for example due to tourism or seasonal labor.

Data considered essential for the purpose of the study are reported in the manuscript. Supportive data are included in Supplementary Tables.

### Standard mortality and standard mortality ratio

In the period 1998–2017, the age composition of the population with a native Dutch background is different from age composition of the population with a western or non-western migration background. For this reason, data of this period from the population with a western or non-western migration background are also presented as age-adjusted incidence per 100,000 of the population according to the age composition of the population with a native Dutch background. This is the Standard Mortality (SM) The SM for persons with a migration background is calculated by the formula

$$S{M}^{(x)}=\mathrm{100,000}\times \frac{D^{(native)}}{N^{(native)}}\times \frac{D^{(x)}}{\sum_i{N}_i^{(x)}\frac{D_i^{(native)}}{N_i^{(native)}}}$$

where ${D}_i^{(x)}$ are the number of deaths in age group *i* and ${N}_i^{(x)}$ the size of the population in age group i with persons with migration background x. The symbols without subscript *i* are the totals over all age groups.

When comparing the periods 2008–2017 with 1998–2007, the age compositions of each of the populations with a native Dutch, western and non-western migration background is different between the two periods. For this reason, data of the age compositions in the period 2008–2017 are also presented as age-adjusted incidences per 100,000 of the population (SM) compared to the population with the same background in the period 1998–2007 (f.e. non-western 2008–2017 vs non-western 1998–2007).

In the period 1998–2017, to allow an alternative comparison between age groups and populations, data are also presented as Standard Mortality Ratio (SMR). The SMR is 1 for drowning of the population with a Dutch background between 1998 and 2017. When comparing the age distribution of the native Dutch population with the western and non-western migration background population, a SMR that is smaller or larger than 1, implicates less or more drowning in the age group of the western and non-western migration background population over the period 1998–2017 compared to the Dutch population.

When comparing the periods 2008–2017 with 1998–2007, the SMR is set at 1 for each population in the period 1998–2007. A SMR over the period 2008–2017 that is smaller or larger than 1, implicates less or more drowning in the populations with the same background. SMR data are included in the additional files as supplementary table to Fig. 2 and Table 2, as well as in the supplementary Tables 3 and 4.

### Statistical analysis

Most data in this study are presented as descriptive data. Statistical analysis has only been applied to analyse differences in migration backgrounds and calculations of trends. T-tests have been applied for differences between the migration background and the comparison between 1998 and 2007 and 2008–2017. For all calculations, *p* < 0.05 was considered statistically significant.

For the trend analysis of the total period 1998–2017, single linear regression analysis has been used. To determine whether there are multiple trends with breakpoints, piecewise regression has been used. Before the breakpoint analysis, outliers identified with Cook’s Distance statistics have been excluded. Programming language R from the statistical package Segmented (https://cran.r-project.org/web/packages/segmented/segmented.pdf) has been used for breakpoint analysis.

### Ethics committee

The data in this study contains aggregated and coded information on age, gender and cause(s) of death, and cannot be traced back to a deceased person. The coded information based on the MDDC is published annually in cause-of-death statistics. This study is a secondary analysis involving no identifiable patient data and no ethical restraints were applicable.

## Results

### The aggregated data

**Fig. 1:**
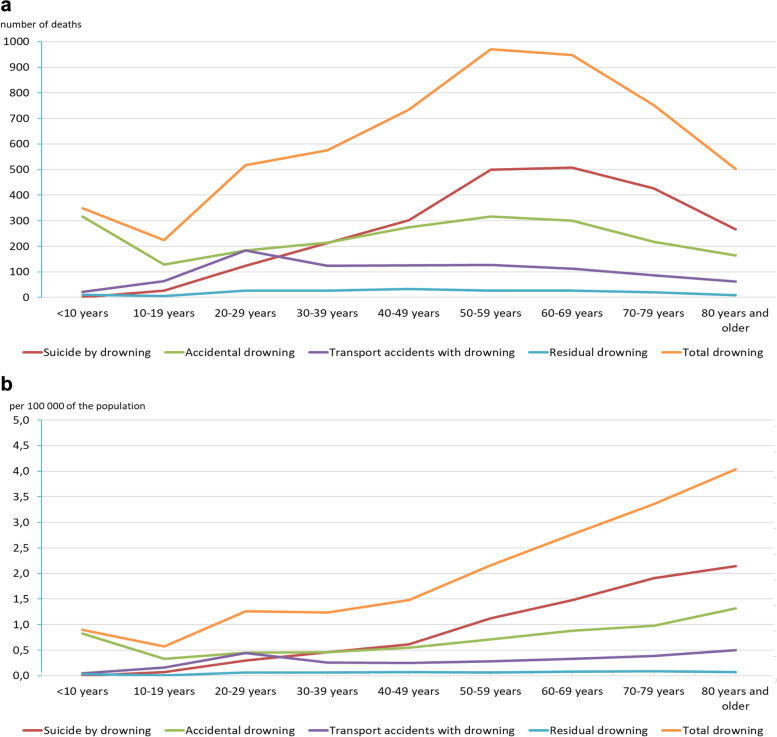
Fatal drowning in the Netherlands 1998–2017. Total number and incidence by age groups and cause of drowning. Additional data is available as Supplementary Table to Figure 1 and Table 1. **a** total numbers by age groups and cause of drowning. **b** incidence per 100,000 of the population by age groups and cause of drowning. Residual drownings: the total of drownings by murder or manslaughter; persons of whom it is unknown whether they either accidentally or intentionally drowned; persons who died more than 30 days after the drowning accident; late effects of drownings; persons who drowned after an epileptic seizure; and who drowned after falling through the ice

5571 people fatally drowned in the Netherlands (incidence: 1.69 per 100,000) between 1998 and 2017. In the total group, the average age is 50.8 years. There are 3914 men (70.3%; average age 48.7 years; incidence 2.40 per 100,000) and 1657 women (29.7%; average age 55.6 years; incidence 1.00 per 100,000). Three main causes (accidental drowning, suicide by drowning and transport accidents with drowning) have been compared and demonstrate different characteristics in terms of total number, incidence per 100,000, age and gender distributions (Fig. 1a and b, Table 1). A residual group with various causes (*n* = 183; 3.3% of the total; 0.06 per 100,000) is included in the overall analysis but not included in the analysis of the three main causes of drowning. In this period, no fatal drowning was recorded as a result of a natural disaster, such as during the several floods that occurred in this period. Details of the aggregated data are available as Supplementary Table to Fig. 1 and Table 1.

**Table 1:** Fatal drowning in the Netherlands 1998–2017. Comparison between the three main causes of drowning. Additional data on age groups is available as Supplementary Table to Figure 1 and Table 1

1998–2017	Suicide by drowning	Accidental drowning	Transport accidents with drowning
Number of drownings	2366	2117	905
Percentage of total drowning	42.5%	39.1%	16.2%
Incidence per 100,000	0.72	0.64	0.28
Male / female	1.44	3.80	3.92
Average age	58.7 yrs	44.5 yrs	45.7 yrs
Age group with the highest number of deaths	60–69 yrs. (*n* = 508) incidence 1.48	0–9 yrs. (*n* = 316) and 50–59 yrs. (*n* = 316) incidence 0.82 and 0.71 resp.	20–29 yrs. (*n* = 183) incidence 0.44
Age group with the lowest number of deaths	0–9 yrs. (*n* = 0)	10–19 yrs. (*n* = 129)	0–9 yrs. (*n* = 21)
Age group with the highest incidence	80+ yrs. (*n* = 267) incidence 2.14	80+ yrs. (*n* = 165) incidence 1.32	80+ yrs. (*n* = 62) incidence 0.50
Age group with the lowest incidence	0–9 yrs. (*n* = 0) incidence 0.00	10–19 yrs. (*n* = 129) incidence 0.33	0–9 yrs. (*n* = 21) incidence 0.05

Values represent absolute numbers with percentages and incidence per 100,000 population stratified by causes of drowning and age groups

### Drowning by migration background

Of the total 5571 drownings, 4892 (87.8%; 1.49 per 100,000) were residents of the Netherlands. Of these, 78.5% (*n* = 3840; 1.46 per 100,000) have a native Dutch background; 8.7% (*n* = 427; 1.43 per 100,000) have a Western migration background and 12.8% (*n* = 625; 1.76 per 100,000) a non-Western migration background. In addition, 679 non-residents drowned (12.2% of the total number). The size of the total population of persons non-residing in the Netherlands is not known and the incidence cannot be calculated. Drowning of residents with various migration backgrounds, and non-residents who drown in the Netherlands, have different characteristics in terms of age, gender, and cause of death.

The SM of all drowned persons with a non-western migration background is almost twice as high as the persons with a western migration or native Dutch background (2.70 vs. 1.44 and 1.46 per 100,000). Similar differences in the SM between the migration backgrounds are observed in suicide and accidental drowning, but not in transport accidents.

**Fig. 2:**
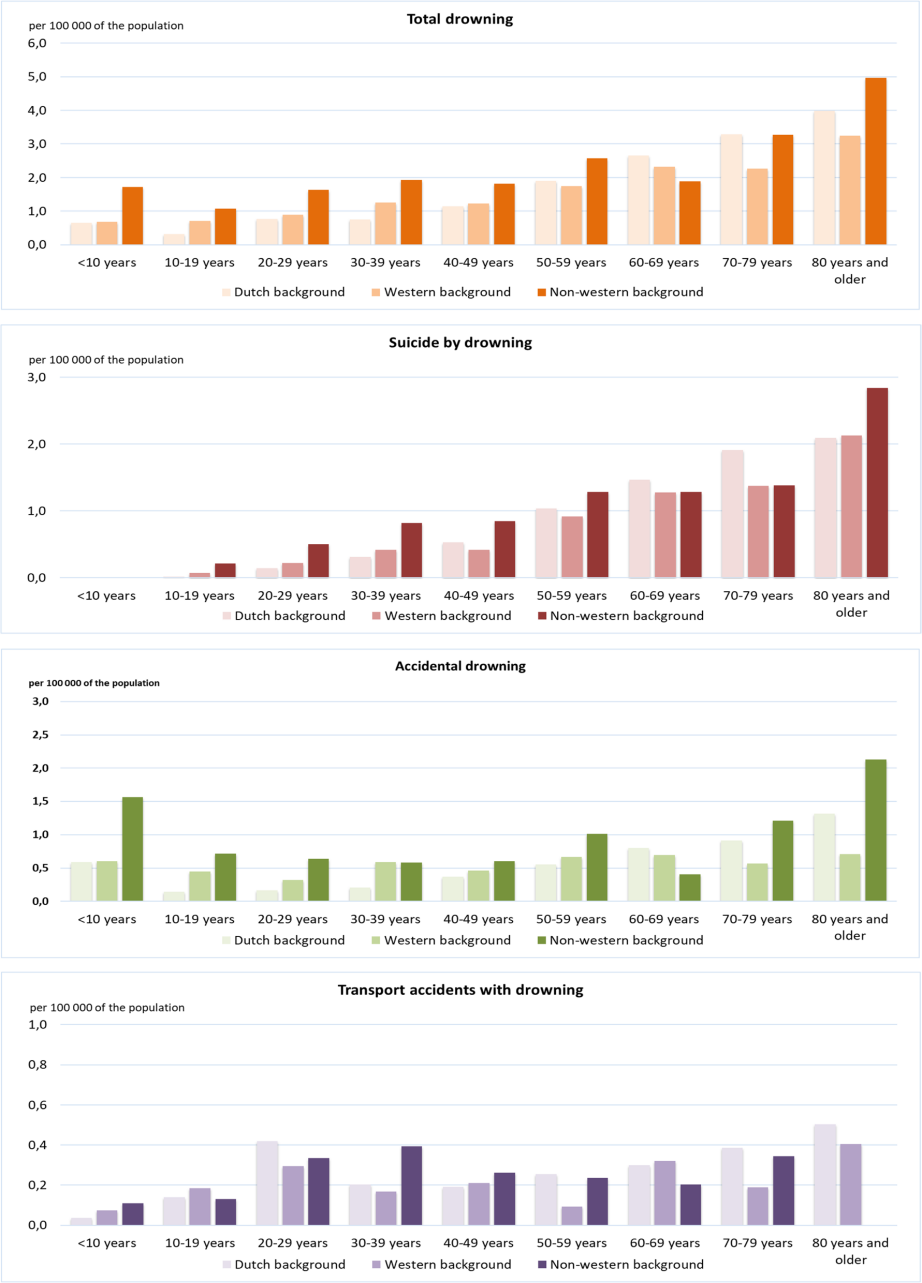
Fatal drowning in the Netherlands 1998–2017. Incidence per 100,000 of the population by cause of drowning, migration background and age group. Additional information on Standard Mortality, Standard Mortality Ratio, Deviation Rate and 95% Confidence Interval is available as Supplementary Table to Figure 2 and Table 2

When comparing the different migration backgrounds by age group, the overall picture is that the incidence by age group is rather similar between persons with native Dutch background and a western migration background until the age of 70 years. The incidence among persons with a western migration background is significantly higher in four age groups and significantly lower in six age groups (Fig. 2 and Table 2).

**Table 2:** Fatal drowning in the Netherlands 1998–2017: Comparison between migration backgrounds and non-residents. Additional data on the incidence per 100,000 population by cause of drowning, migration background and age is available as Supplementary Table to Figure 2 and Table 2

	Residents of the Netherlands			Non-residents of the Netherlands
1998–2017	Native Dutch background	Western background	Non-western background	
**Total drowning**				
Number of drownings	3840 (68.9%)	427 (7.7%)	625 (11.2%)	679 (12.2%)
Incidence per 100,000	1.46	1.43	1.76	–
Standard Mortality	1.46	1.44	2.70*	–
Average age at death	55.6 yrs	50.2 yrs	32.5 yrs	40.7 yrs
Male / female	2.1	2.2	3.3	4.9
**Suicide by drowning**				
Number of drownings	1803	185	209	169
Percentage of total drownings	47.0%	43.3%	33.4%	24.9%
Incidence per 100,000	0.68	0.62	0.59	–
Standard Mortality	0.68	0.62	1.16*	–
Average age at death	61.9 yrs	58.4 yrs	42.6 yrs	44.3 yrs
**Accidental drowning**				
Number of drownings	1257	163	298	399
Percentage of total drownings	32.7%	38.2%	47.7%	58.8%
Incidence per 100,000	0.48	0.55	0.84	–
Standard Mortality	0.48	0.57*	1.15*	–
Average age at death	50.9 yrs	43.5 yrs	25.3 yrs	38.8 yrs
**Transport accidents with drowning**				
Number of drownings	647	60	87	111
Percentage of total drownings	16.8%	14.1%	13.9%	16.3%
Incidence per 100,000	0.25	0.20	0.24	–
Standard Mortality	0.25	0.20	0.28	–
Average age at death	48.1 yrs	43.7 yrs	32.9 yrs	42.0 yrs

--- The total non-resident population is not known and therefore the incidence and the Standard Mortality cannot be calculated

* The Standard Mortality (SM) is significantly higher compared to the other migration backgrounds (t-test: *p* < 0.05)

When comparing between persons with a native Dutch background and a non-western background, the differences are much larger. The incidence in persons with a non-western background is significantly higher in 16 age groups and lower in two age groups. The largest difference in the total group of drowning is found in the groups 0–9 years, mainly caused by the higher incidence of accidental drowning (0.59 versus 1.56 per 100,000); this is 2.7 times higher than among persons with a native Dutch background. The large difference in the age 10–19 years is caused by the higher incidence of drowning by suicide (0.02 vs. 0.21 per 100,000) and accidental drowning (0.14 versus 0.71 per 100,000). This is 10.8 and 5.1 times higher respectively than among persons with a native Dutch background.

Details of the data related to migration background are available as a Supplementary Table to Fig. 2 and Table 2.

### Trends

**Fig. 3:**
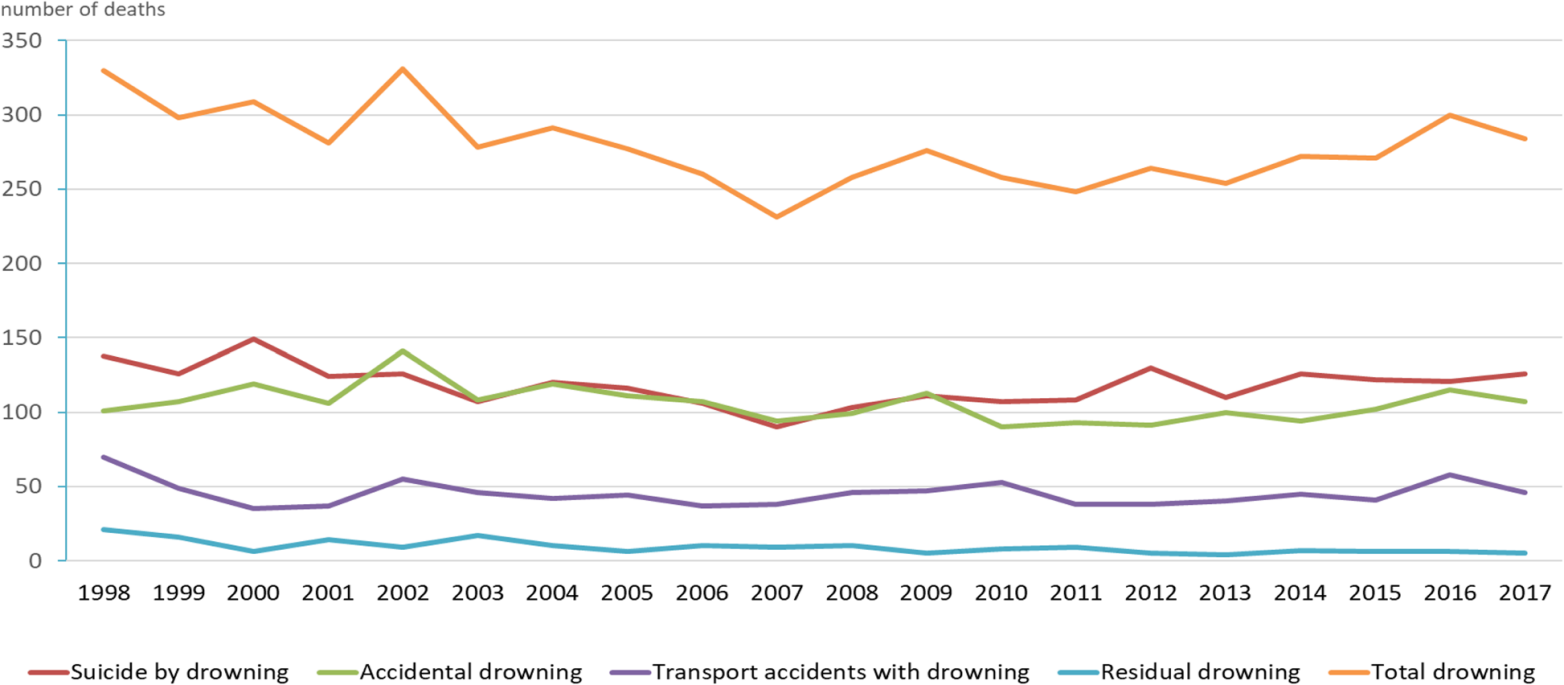
Fatal drowning in the Netherlands 1998–2017; trend of total number by year and cause of drowning. Additional information on Incidence, Standard Mortality, Standard Mortality Ratio, Deviation Rate and 95% Confidence is available as Supplementare Table to Figure 3. Residual drownings: the total of drownings by murder or manslaughter; persons of whom it is unknown whether they either accidentally or intentionally drowned; persons who died more than 30 days after the drowning accident; late effects of drownings; persons who drowned after an epileptic seizure; and who drowned after falling through the ice

The highest absolute number of drowning in the total group occurred in 2002 (331; 2.05 per 100,000), the lowest in 2007 (231; 1.41 per 100,000 (Fig. 3). Additional data on the absolute numbers is available in Supplementary Table to Fig. 3.

**Fig. 4:**
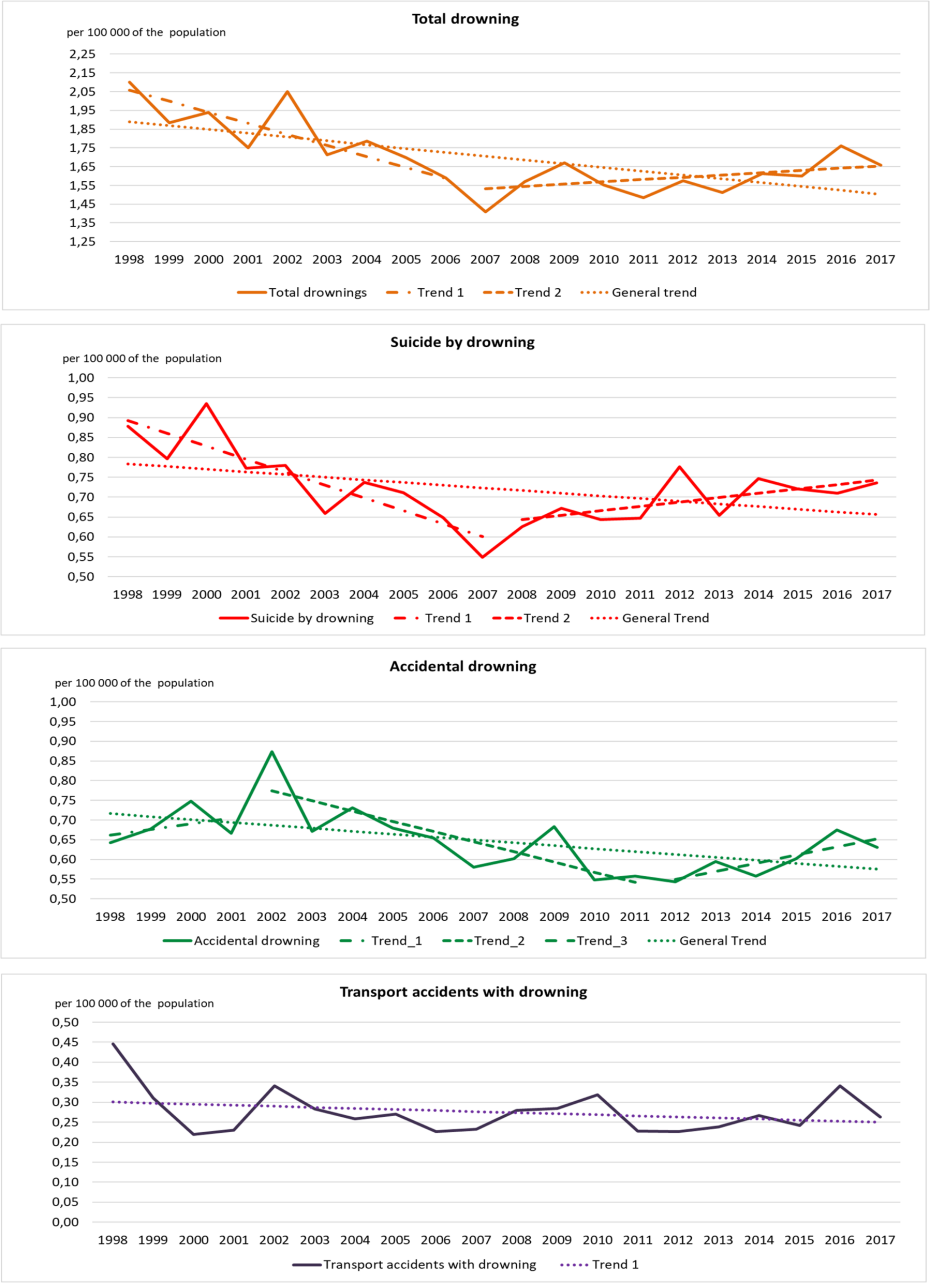
Trends of fatal drowning in the Netherlands 1998–2017; incidence per 100,000 of the population by cause of drowning; single regression analysis and breakpoint analysis, Additional information of the breakpoint analysis is available in Supplementary Table to Figure 4

In terms of the incidence, over the complete period of the study (1998–2017), there is a decreasing trend of all drowning (adjusted R^2^: 0.40), suicidal drowning (adjusted R^2^: 0.15), accidental drowning (adjusted R^2^: 0.26) and drowning due to transportation (adjusted R^2^: 0.03) (Fig. 4).

Fig. 4 also shows a breakpoint in the trends of the incidence for all drowning in 2007 (adjusted R^2^: 0.70; before 2007: level 85.3, slope − 4.2%; after 2007: level - 23.6, slope 1.3%). For drowning by suicide, a breakpoint is identified in 2008 (adjusted R^2^: 0.66; before 2008: level 65.7, slope − 3.3%; after 2008: level − 21.5, slope 1.1%). For accidental drowning there are 2 breakpoints. In 2002 (R^2^: 0.55; before 2002: level − 27.7, slope 1.4%; after 2002: level 52.4, slope − 2.6%) and in 2012 (before 2012: level 52.4, slope − 2.6%; after 2012: level − 40.9, slope 2.1%). There is no breakpoint identified for drowning due to transportation (R^2^: 0,51, level 3.1, slope − 1.0%). Details of the trends and breakpoints are available as Supplementary Table to Fig. 4.

When comparing the SM and SMR of the total group and the three causes of drowning between 2008 and 2017 (*n* = 2658) with the previous decade 1998–2007 (*n* = 2886), a significant decrease can be observed. The share of female drowning victims did not significantly change between the two periods (Table 3) Furthermore, the SM and SMR of all causes of drowning for residents with native Dutch and non-western backgrounds decreases significantly in the recent decade. The same pattern is identified for suicide by drowning and accidental drowning. There was no significant decrease for people with a western background, both regarding the total group and the 3 causes studied (Table 4).

**Table 3:** Fatal drowning in the Netherlands; comparison between 1998 and 2007 and 2008–2017 by cause of drowning and age group. The mortality ratio of 2008–2017 is age-adjusted (SM) based on the population 1998–2007. Additional information on Standard Mortality, Standard Mortality Ratio, Deviation Rate and 95% Confidence Interval is available as Supplementary Table 3

	1998–2007	2008–2017
	Standard Mortality (SM)	
**Total drowning**		
Number of drownings	2886	2685
Incidence per 100,000	1.79	1.60
Standard Mortality	1.79	1.51**
Average age at death	48.9 yrs	52.8 yrs
Male / female	2.3	2.4
**Suicide by drowning**		
Number of drownings	1202	1164
Incidence per 100,000	0.75	0.69
Standard Mortality	0.75	0.63**
Average age at death	57.9 yrs	59.4 yrs
Male / female	1.4	1.5
**Accidental drowning**		
Number of drownings	1113	1004
Incidence per 100,000	0.69	0.60
Standard Mortality	0.69	0.58**
Average age at death	41.9 yrs	47.3 yrs
Male / female	3.8	3.7
**Transport accidents with drowning**		
Number of drownings	453	452
Incidence per 100,000	0.28	0.27
Standard Mortality	0.28	0.26
Average age at death	48.9 yrs	52.8 yrs
Male / female	3.7	4.0

** Standard Mortality (SM) in the period 2008–2017 is significantly lower compared to 1998–2007 (t-test: *p* < 0.05)

**Table 4:** Fatal drowning in the Netherlands; comparison between 1998 and 2007 and 2008–2017 by migration background. The incidence of 2008–2017 is age-adjusted (Standard Mortality) based on the population 1998–2007 of the group with the same background. Additional information on Standard Mortality, Standard Mortality Ratio, Deviation Rate and 95% Confidence Interval by cause of drowning and migration background is available as Supplementary Table 4

	1998–2007	2008–2017
	Standard Mortality (SM)	
**Total drownings**		
Native Dutch background	1.55	1.25**
Western background	1.50	1.34
Non-western background	1.99	1.54*
**Suicide by drowning**		
Native Dutch background	0.71	0.59**
Western background	0.65	0.56
Non-western background	0.60	0.50**
**Accidental drowning**		
Native Dutch background	0.52	0.41**
Western background	0.57	0.52
Non-western background	1.00	0.75**
**Transport accidents with drowning**		
Native Dutch background	0.26	0.22**
Western background	0.19	0.22
Non-western background	0.28	0.21**

** Standard mortality (SM) in the period 2008–2017 is significantly lower compared to 1998–2007 (t-test: *p* < 0.05)

**Fig. 5:**
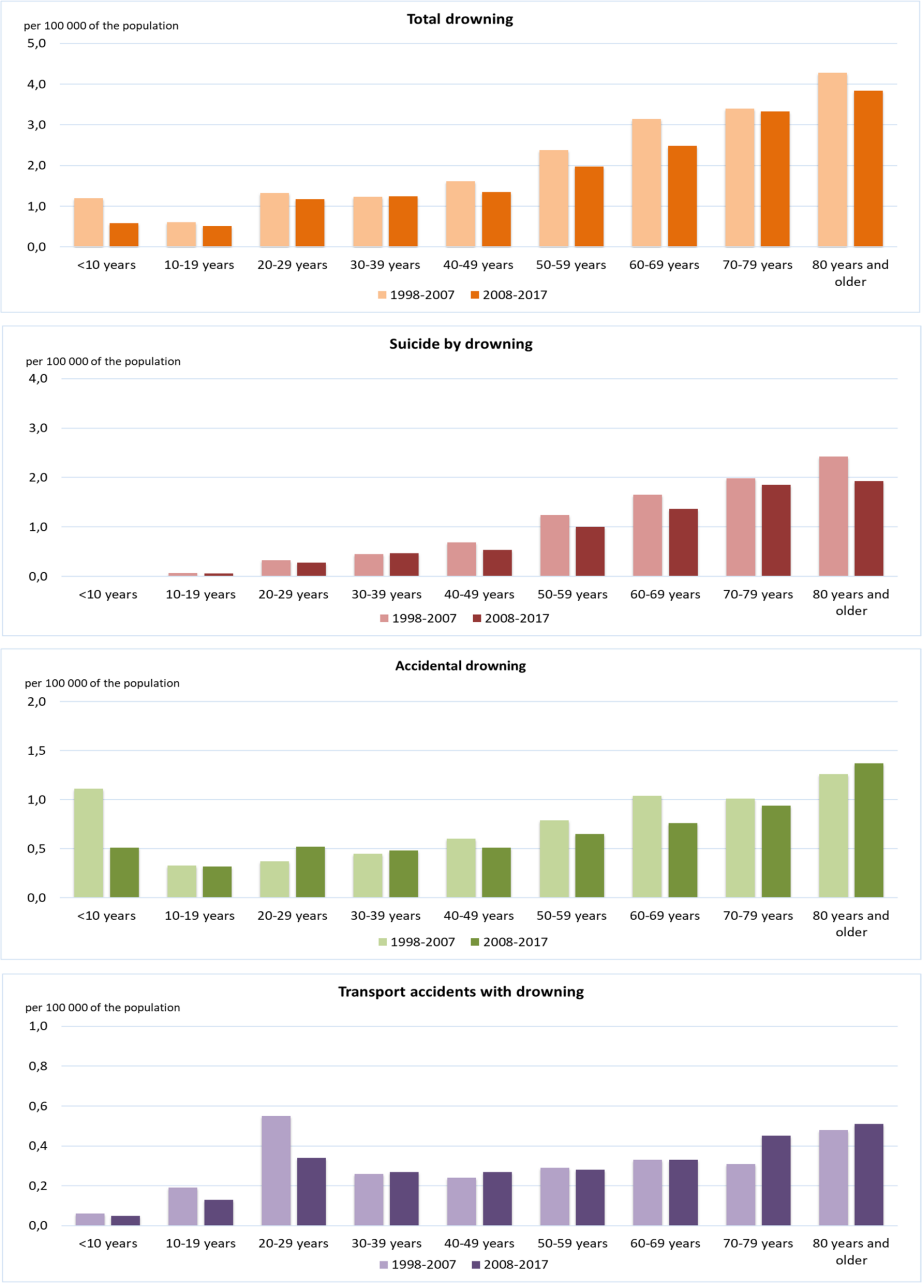
Fatal drowning in the Netherlands, comparison between 1998 and 2007 and 2008–2017: by cause of drowning and age group. Additional information on Standard Mortality, Standard Mortality Ratio, and 95% Confidence Interval is available in Supplementary Table 3

Comparing the periods 1998–2007 and 2008–2017 in terms of age-related and cause-related incidences, a scattered pattern emerges (Fig. 5). There are 2 significantly increased incidences: accidental drowning among 20 to 29-year-olds and drowning in transport accidents among 70–79-year-olds. For all other causes and ages, the incidence has remained the same or is significantly reduced. For the total group, significant reductions are observed for the 0–9 and 40–69 years old. The drop in the share held by the group 0–9 years is mainly due to a decline in accidental drowning in this age group. The decline in the group 40–69 years is largely caused by a significant decline in suicide by drowning and accidental drowning in that age group. Details of the trend data are available as Supplementary Table to Fig. 5 and as Supplementary Tables 3 and 4.

## Discussion

To our best knowledge, this is the first study based on unrestricted direct access to all existing data in the national death statistics that was able to depict an as-complete-as-possible profile of the national drowning mortality. Historically experienced problems to obtain these data were most of all solved by direct cooperative involvement of the department in charge of death statistics at the CBS.

The formal, officially recognised and often cited, total number of deaths by drowning in the Netherlands between 1998 and 2017 is 1718; about 85 fatal drownings per year [36, 37]. The number is based on accidental drowning by Dutch residents. To obtain the complete number of drowning, this study was extended to all causes of drowning and at the same time to drowning by non-residents. In this way, the aggregated and complete profile of drowning could be identified, which included 5571 drownings; approximately 275 fatal drownings per year. The currently published incidence of accidental drowning by Dutch residents is 0.52 per 100,000 of the population. With this method, the incidence increases to 1.69 per 100,000 of the population, an increase of almost 350%.

For over two decades, there have been discussions about the real number of fatal and non-fatal drowning worldwide. Already at the end of the previous century, several publications demonstrated concern that the common way of reporting drowning statistics underestimates the actual numbers [3–6, 38]. For example, the first World Congress on Drowning was being prepared with the motivation that “each year an estimated number of 150,000 people die from drowning. At least the same number of victims (but probably 2-20 times that number) are admitted to hospitals for observation and treatment” [39]. This number appears to be an underestimation when, in 1999, the WHO published a report in which the total burden of drowning is estimated to affect 1.1 million people [40]. The report identifies drowning as the third largest cause of death by unintentional injury and estimates that drowning constitutes 8% of the injury deaths in the world. The same report acknowledges that reliable numbers are missing from many countries, and particularly in low and middle-income countries underestimations are reported due to imprecise data. In 2004, the United National International Children’s Emergency Funds (UNICEF) together with The Alliance for Safe Children (TASC) observes that most drowning victims are never brought to a hospital and therefore not included in the information systems of low and middle-income countries that report on mortality) [41]. In 2008, the WHO together with UNICEF stated that the quality of the mortality data of injuries tends to be weakest where the problems are greatest [42]. In 2012, UNICEF together with The Working Group on Child Drowning in Low- and Middle-Income countries published a report on child drowning that attempts to overcome earlier encountered structural, cultural, societal, legal and financial problems in data collection and data coding at the national level by capturing data at local level by means of verbal autopsies: interviews in selected households and communities. They concluded that there is 58–330% of underreporting [43]. In 2014, the WHO Global Report on Drowning pointed out that inaccurate coding and poor data collection systems obscure the full scale of global drowning [1]. In 2017, another study concluded that the specificity, and thus quality, in drowning method reporting varied across countries [38]. A recent drowning publication based on the Global Burden of Disease 2017 study estimates that ICD codes for accidental drowning account for 40–50% of all drowning in high-income countries [2].

In the same period, from 2002 onwards, researchers from several countries have tried to compile all-inclusive and complete data on fatal drowning. These population-based studies from France [44], the Netherlands [45], Australia, Canada and New Zealand [20, 26, 27, 46, 47], Tanzania [48], China [24], Fiji [49], Philippines [14], Iran [50], and Czech Republic [51] conclude that 3 to 13 data sources need to be merged in order to obtain a complete picture of the fatal national drowning data. Each of these studies recognises major problems. These problems include data collection limited to a restricted period of the year, poor quality of the definitions used, nomenclature, categorisation, coding and coding hierarchy of the data, availability of data that are only retrievable after a long complex and costly procedure, poor compliance of those who have to provide the data information to the system and low priority to the issue of drowning. Another cause of incomplete data is the result of the large portion of drowning of undetermined intent or open verdict by forensic pathologists and forensic doctors about the cause of drowning [52–54].

Most of these studies recognise that by only including accidental drowning, large numbers of drowning are excluded from the official statistics. For this reason, to obtain a more complete picture, a study from the Philippines includes transportation accidents (V90–92) and natural disasters (X-38), and concludes that the incidence of drowning in this way increases from 6.0 to 8.5 per 100,000 population [14]; a study in Tanzania, including the full range of drowning related ICD-10 codes, concludes that the drowning mortality is 16% higher than previously considered [47]. Australian studies including multiple drowning codes and triangulation found an increase of 40% [26, 27]. Using a capture-recapture method, the mortality from drowning increased from 4.5 to 8.3 per 100.000 in 2 Northern-Iran provinces [50].

The information below provides more detailed information on causes, demographical characteristics and trends from this study, as well as their relationship with international data. All these data had not been available before; they provide new and unrecognised aspects of the fatal drowning profile that may be used for public health interventions.

### Suicide

This study shows that the largest contribution in terms of absolute numbers (*n* = 2366) and incidence (0.72 per 100,000) is made by suicidal drowning. Previous studies on suicidal drowning were concerned with underreporting of drowning by suicide and it being misclassified as accidental drowning [55–57] or transportation accidents [4]. We believe that there is very limited underreporting in this study because data on the death certificate are carefully compared with the information provided in the court document by the PPO.

The portion of 42.5% of all drowning by suicide, found in this study, is extremely high. A recent study of suicidal drowning in 32 OECD countries (2012–2014) ranks the Netherlands second-high [58]. Other national study on suicidal drowning report a portion between 7.2 and 31% [32, 51–53, 59–62]. The incidence of suicide by drowning in all studies is consistently highest in the age groups of 70 years and above [52, 53, 56, 59, 63, 64]. Most studies find that women are more likely to commit suicide by drowning than men, although there are significant gender differences between countries and regions [52, 53, 56, 62–66]. Drowning seems also not to be the preferred method of suicide for young people [67]. When comparing between suicides by persons with a native Dutch background and those by persons with a non-western migration background, the suicide rate is statistically higher in the age groups 10–49 years and non-statistically higher in the age groups 50–59 years and 80 years and older. The lower incidence of suicides by non-western inhabitants in the Netherlands has been reported before [68, 69].

Of all suicides in the Netherlands, approximately 7.0% is by drowning [62]. In a database that includes 160,460 suicides in 16 European countries over a 5-years period, drowning accounted for 3% of all male and 7.8% of all female suicides; the highest percentage was 25.5% in Irish women and the lowest percentage was 0.3% for Estonian men [63]. Several other non-European studies [54, 56, 59, 64] and one systematic review [62] also show that of all suicide methods, drowning constitutes a small portion. Several recent studies have detected a decreasing trend in drowning by suicide [54, 62, 64].

A number of studies have tried to explain the differences in suicide by drowning between countries by length of coastline or shoreline, distance to water, “blue spaces” (visible bodies of fresh and salt water), and culturally influenced gender-specific self-destructive behaviour [63, 70–72]. In none of the studies, a correlation could be detected. A more likely explanation of the large proportion of drowning by suicide in the Netherlands might be the omnipresent accessibility to water, the lack of access to other means such as firearms, railway suicide prevention programmes and the robust regulations for the prescription of medications [73, 74].

### Accidental drowning

This study confirms a recently described observation in studies from high-income countries, observing that the traditionally highest incidence of accidental drowning in the 0–9 years old group is gradually replaced by the highest incidence in the age categories above 50 years of age [2, 26, 75], including South Africa [76], Canada [77], Sweden [61, 78] and China [24]. There may be several reasons for this change, including more leisure time and exposure to water for those who are still fit and healthy at this age, or on the other end, weaker health and condition that causes a minor incident to have a fatal outcome. In all accidental drowning studies, the majority of the victims are male, although the male: female ratio may vary a lot between countries, ages, and locations [23, 26, 76, 79, 80]. Some studies showed relative higher rates of accidental female drowning in bathtubs [8] or in female indigenous people [46].

### Transportation

It should not come as a surprise that transportation drowning is common in a country with high population density and abundant waterways often immediately next to roads (330,000 km of ditches, 6500 km of canals, 6200 km of streams and 650 km of rivers) [81]. In this study, land and water transportation accidents have been combined and result in 16.2% of all drowning deaths. A few other studies on transportation drowning describe that between 5.6 and 15.8% of all fatal drowning is related to transportation [53, 77, 82, 83]. Because drowning during transport is often coded as transportation death and not as drowning death, other sources than ICD codes had to be used to understand the incidence of transportation drowning [5, 84]. The causes vary and include recreational boating [85, 86], fishing [87–89], public transport by water [84, 88], high speed crafts [90], cruise ships [91] as well as snow mobiles [23, 92, 93], submerged cars [94–97], and cars trapped in floods [26, 98]. The Dutch Institute for Road Safety Research (Stichting Wetenschappelijk Onderzoek Verkeersveiligheid: SWOV) reports traffic accidents, including traffic accidents that end in water. Unfortunately, the data are almost all based on police reports and do not allow an overall picture of the total number of transport accidents that result in fatal drowning [99–101].

### Ethnicity and non-residents

Another significant observation from this study, and reported previously [102, 103], is that a significant portion (41%) of all accidental drowning in the Netherlands concern inhabitants with a western migration background (8%), a non-western migration background (14%), and non-residents (19%). The SM of persons with a non-western migration background is almost twice as high as among persons with a western migration background or native Dutch background (2.70 vs 1.44 and 1.46 resp. per 100,000). It can be assumed that many of them are not yet familiar with the omni-presence of water and the typical characteristics such as low temperature, current, and bottom profiles. In addition, peer pressure (children with a migration background with lower swimming skills follow friends with a Dutch background with better swimming skills in risky behaviour) seems a relevant factor [104–106]. The incidence of accidental drowning among persons with a western background is the same as for persons with a native Dutch background (0.5 and 0.6 resp. per 100,000). These incidence figures of accidental drowning are in line with the incidence of drowning mortality in the western part of Europe, northern American countries and Australia [20, 22, 107–110]. On the other hand, the incidence of accidental drowning for Dutch inhabitants with a non-Western background is significantly higher (1.2 per 100,000); this comes close to international statistics of non-Western countries [2]. It is not possible to calculate the incidence of drowning of people who are not registered in the Netherlands. The total number of accidental drowning fatalities is higher among non-residents (*n* = 399) than among non-western residents (*n* = 298). The difference in the total number is smaller due to fewer suicides (679 vs 625). Non-residents are often ignored in national drowning statistics and when included, often reported by countries and islands where the beaches and pools are populated by tourists [15, 19, 111–115]. Some data on non-residential drowning can be extracted from annual national lifesaving reports [116, 117]. Non-residents who drown in the Netherlands are often foreign workers, asylum seekers and, for example, tourists who drown in canals after a visit to a bar [118].

### Trends

SM and SMR in the first decade (1998–2007) compared to the second decade (2008–2017), as well as decreasing trend when a single regression analysis was applied over the period 1998–2017, show a decline regarding all causes, most age groups and residents with native Dutch and non-western backgrounds. The reduction of accidental drowning numbers in the Netherlands would have been well in line with statistics from most high-income countries around the world, where a consistent and on-going decreasing trend can be observed. This includes significant decreases in 9–14 years old children in Europe between 1990 and 2016: a total decrease of 64.4% and in children aged 5–9 years of 72% [22]. Similar findings are reported in 33 European countries [119], and Australia [120]. The decrease, with rates that vary between 5 and 80%, is also reported in studies initiated in low and middle-income countries or based on the Global Burden of Disease registrations (GBD) [2, 24, 38, 77, 121–124]. This decreasing trend in accidental drowning is speculatively explained by better socioeconomic situations, improved infrastructure planning such as piped water supply resulting in less frequent and less easy access to water, mandatory education, safer swimming and bathing facilities, aquatic safety education and swimming lessons [10, 22, 24, 125–127). Notably more urbanisation is hypothesised to be a major factor, as this decreases the access to open water, causes shifts towards indoor activities and entertainment and reduces income differences [2, 24, 28]. A decreasing trend in drowning by suicide had also been reported [66, 128].

In addition to the comparison between two periods and a single regression analysis, also a breakpoint analysis has been performed. This resulted in the remarkable observation that, within the decreasing trend over the full period under investigation, there is an upward trend in the total number of drowning after 2007, in suicidal drowning after 2009 and in accidental drowning after 2012. These recent upward trends were missed in the trend analysis over the full period and contradict the general impression that the incidence of drowning in the Netherlands is stable or decreasing. There is no explanation for this observation at this moment.

### Limitations

This study has certain limitations. First of all, the focus of data reporting and analysis is on a small selection of demographic data. The inclusion of more details may have provided different clues for a better understanding of the national burden of drowning such as body of water, mechanism of drowning, and geographic, seasonal and economic data [121]. These data are available and may be reported in the future. Also, data on non-fatal drowning victims recorded by aquatic rescue organisations and patients admitted to emergency departments and intensive care units will enhance the perspective on the issue [3, 46, 129–131]. Although categorisation systems for clinical studies are available which report fatal and non-fatal drowning data in a standardised way, these systems are not suitable for demographic or epidemiological studies [4, 127, 132, 133].

Very likely, not all Dutch persons who drowned outside the Netherlands have been included or may have been included with less accurate data.

It may have been interesting to compare the data on age and migration background before and after the various breakpoints for each group of drowning. For reasons of consistent reporting and to avoid an overload of data, we have compared two equal periods (1998–2007 vs 2008–2017). Seasonal effect, net drift, local drift [24], piecewise analysis or joint point trend analysis could have been included to further explore if any other trend could be detected.

It was decided to not include such information because the results of these analyses would substantially increase the length of the paper and distract from the objective of this study.

## Conclusion

Reporting on the total drowning burden is a much-needed area of study with underreported gaps in the literature. This paper addresses these gaps and provides detailed analysis based on an inclusive dataset. The actual incidence of fatal drowning found in this study is 350% higher than the public available number of drowning. 39.1% of the drownings are accidental drowning by residents, which in general is considered the official drowning estimate in the presentation of national drowning statistics. Drowning by suicide and drowning by persons over the age of 50 are leading in the drowning statistics of the Netherlands, 12.2% are non-residents. Although the total number of drowning remains more or less the same, the incidence of drowning and comparing 1989–2007 with 2008–2017, show a decreasing trend over a period of 20 year. An additional breakpoint analysis however observed several recent upwards trends.

The study emphasises once again that national drowning statistics, comparisons between national drowning statistics based on accidental drowning, and trend analysis need to be interpreted cautiously. Countries may have different proportions of drowning by suicide, accidental, transport and disasters and therefore the proportion of victims not included in statistics of accidental drowning. In-depth studies, knowledgeable trend analysis and looking beyond routinely collected and reported data are required to accurately investigate the burden of drowning and to understand the large variety in the numbers of deaths by drowning among countries. This study underlines the importance of recently (April 2021) passed United Nation resolution on drowning that advises “*to aggregate all drowning mortality data into national estimates*” as one of the actions to address this largely under-recognised public health problem [134].

## Supplementary Information

**Additional file 1.** Supplementary Table to Figure 1 and Table 1. Fatal drowning in the Netherlands 1998–2017; total number and incidence per 100,000 of the population by cause of drowning and age group. Supplementary Table to Figure 1a. Total number of fatal drowning in the Netherlands 1998–2017; by cause of drowning and age group. Supplementary Table to Figure 1b. Incidence per 100,000 of the population of fatal drowning in the Netherlands 1998–2017; by cause of drowning and age group.

**Additional file 2.** Supplementary Table to Figure 2 and Table 2. Fatal drowning in the Netherlands 1998–2017: Comparison between migration backgrounds and non-residents. Additional information on Standard Mortality, Standard Mortality Ratio, Deviation Rate and 95% Confidence Interval by cause of drowning, migration background and age group.

**Additional file 3.** Supplementary Table to Figure 3. Trend of fatal drowning in the Netherlands 1998–2017; by cause of drowning: trend of total number and of incidence per 100,000 of the population by year and cause of drowning.

**Additional file 4.** Supplementary Table to Figure 4. Trends of fatal drowning in the Netherlands 1998–2017; incidence per 100,000 of the population by cause of drowning; single regression analysis and breakpoint analysis. Additional information of the breakpoint analysis.

**Additional file 5.** Supplementary Table 3. Fatal drowning in the Netherlands 1998–2017; comparison between 1998 and 2007 and 2008–2017 by cause of drowning and age group. The mortality ratio of 2008–2017 is age-adjusted (SM) based on the population 1998–2007. Additional information on Standard Mortality, Standard Mortality Ratio, Deviation Rate and 95% Confidence Interval.

**Additional file 6.** Supplementary Table 4. Fatal drowning in the Netherlands 1998–2017; comparison between 1998 and 2007 and 2008–2017; Additional information on Standard Mortality, Standard Mortality Ratio, Deviation Rate and 95% Confidence Interval by cause of drowning and migration background.

## Data Availability

By law, information from individual records is not allowed to leave the offices of the CBS as this may result in the identification of individual persons. For this reason, the original datasets generated and analysed during the current study are not publicly available. The public available data sets are available at. https://www.cbs.nl/en-gb/our-services/methods/definitions and https://www.cbs.nl/en-gb/our-services/methods/surveys/korte-onderzoeksbeschrijvingen/causes-of-death-statistics Tables and figures that are at the basis of the current manuscript are available from the corresponding author on reasonable request.
