# Supplementary material for: Fatal drowning statistics from the Netherlands – an example of an aggregated demographic profile
Source: BMC Public Health. 2022 Feb 17;22:339. doi: 10.1186/s12889-022-12620-3 (PMC8851711; doi:10.1186/s12889-022-12620-3)
Supplement: Supplementary file 1 — Additional file 1. Supplementary Table to Figure 1 and Table 1. Fatal drowning in the Netherlands 1998–2017; total number and incidence per 100,000 of the population by cause of drowning and age group. Supplementary Table to Figure 1a. Total number of fatal drowning in the Netherlands 1998–2017; by cause of drowning and age group. Supplementary Table to Figure 1b. Incidence per 100,000 of the population of fatal drowning in the Netherlands 1998–2017; by cause of drowning and age group. [file 12889_2022_12620_MOESM1_ESM.pdf]

Supplementary Table to Figure 1 and Table 1. Fatal drowning in the Netherlands 1998-2017; total number and incidence per 100,000 of the population by cause of drowning and age group
Supplementary Table to Figure 1a. Total number of fatal drowning in the Netherlands 1998-2017; by cause of drowning and age group
Supplementary Table to Figure 1b. Incidence per 100,000 of the population of fatal drowning in the Netherlands 1998-2017; by cause of drowning and age group

Supplementary Table to Figure 1 and Table 1. Fatal drownings in the Netherlands 1998-2017; total number and incidence per 100,000 of the population by cause of drowning and age group

	Suicide by drowning			Accidental drowning			Transport accidents with drowning			Residual drowning			Total drowning		
	Males	Females	Total	Males	Females	Total	Males	Females	Total	Males	Females	Total	Males	Females	Total
	Number of deaths														
<10 years	0	0	0	219	97	316	18	3	21	7	4	11	244	104	348
10-19 years	18	8	26	108	21	129	46	18	64	3	2	5	175	49	224
20-29 years	101	23	124	167	17	184	147	36	183	23	3	26	438	79	517
30-39 years	157	55	212	199	16	215	100	23	123	23	3	26	479	97	576
40-49 years	185	117	302	236	38	274	104	21	125	26	7	33	551	183	734
50-59 years	273	227	500	245	71	316	94	33	127	18	9	27	630	340	970
60-69 years	285	223	508	240	60	300	95	18	113	22	4	26	642	305	947
70-79 years	210	217	427	156	62	218	68	19	87	14	6	20	448	304	752
80 years and older	157	110	267	99	66	165	46	16	62	5	4	9	307	196	503
Total	1386	980	2366	1669	448	2117	718	187	905	141	42	183	3914	1657	5571
	Per 100 000 of the population														
<10 years	0,00	0,00	0,00	1,11	0,52	0,82	0,09	0,02	0,05	0,04	0,02	0,03	1,24	0,55	0,90
10-19 years	0,09	0,04	0,07	0,53	0,11	0,33	0,23	0,09	0,16	0,01	0,01	0,01	0,87	0,25	0,57
20-29 years	0,49	0,11	0,30	0,80	0,08	0,45	0,71	0,18	0,44	0,11	0,01	0,06	2,11	0,39	1,26
30-39 years	0,67	0,24	0,46	0,85	0,07	0,46	0,43	0,10	0,26	0,10	0,01	0,06	2,05	0,42	1,24
40-49 years	0,74	0,48	0,61	0,95	0,15	0,55	0,42	0,09	0,25	0,10	0,03	0,07	2,21	0,75	1,48
50-59 years	1,21	1,02	1,12	1,08	0,32	0,71	0,42	0,15	0,28	0,08	0,04	0,06	2,79	1,53	2,16
60-69 years	1,68	1,29	1,48	1,42	0,35	0,88	0,56	0,10	0,33	0,13	0,02	0,08	3,79	1,76	2,77
70-79 years	2,09	1,76	1,91	1,55	0,50	0,98	0,68	0,15	0,39	0,14	0,05	0,09	4,46	2,47	3,36
80 years and older	3,75	1,33	2,14	2,37	0,80	1,32	1,10	0,19	0,50	0,12	0,05	0,07	7,34	2,37	4,04
Total	0,85	0,59	0,72	1,02	0,27	0,64	0,44	0,11	0,28	0,09	0,03	0,06	2,40	1,00	1,69
