# Supplementary material for: Fatal drowning statistics from the Netherlands – an example of an aggregated demographic profile
Source: BMC Public Health. 2022 Feb 17;22:339. doi: 10.1186/s12889-022-12620-3 (PMC8851711; doi:10.1186/s12889-022-12620-3)
Supplement: Supplementary file 2 — Additional file 2. Supplementary Table to Figure 2 and Table 2. Fatal drowning in the Netherlands 1998–2017: Comparison between migration backgrounds and non-residents. Additional information on Standard Mortality, Standard Mortality Ratio, Deviation Rate and 95% Confidence Interval by cause of drowning, migration background and age group. [file 12889_2022_12620_MOESM2_ESM.pdf]

Supplementary Table to Figure 2 and Table 2. Fatal drowning in the Netherlands 1998-2017: Comparison between migration backgrounds and non-residents. Additional data on the incidence per 100,000 population by cause of drowning, migration background and age. For age-specific data incidence and SM are identical.

Supplementary Table to Figure 2 and Table 2

Fatal drownings in the Netherlands 1998-2017; per 100,000 of the population by cause of drowning, migration background and age group

	Dutch background				Western background					Non-western background				
	SM	SMR	SD	CI (95%)	SM	SMR	SD	CI (95%)	sign.	SM	SMR	SD	CI (95%)	sign.
**Total drowning**														
<10 years	0,65	1	0,07	0,86-1,14	0,68	1,04	0,25	0,56-1,52		1,72	2,54	0,25	2,05-3,04	*
10-19 years	0,31	1	0,10	0,80-1,20	0,70	2,25	0,52	1,24-3,26	*	1,07	3,42	0,42	2,59-4,24	*
20-29 years	0,77	1	0,07	0,87-1,13	0,89	1,15	0,19	0,77-1,52		1,63	2,11	0,20	1,71-2,51	*
30-39 years	0,76	1	0,06	0,88-1,12	1,26	1,66	0,21	1,24-2,08	*	1,93	2,55	0,23	2,10-3,00	*
40-49 years	1,15	1	0,05	0,91-1,09	1,22	1,06	0,14	0,79-1,34		1,82	1,58	0,17	1,25-1,90	*
50-59 years	1,91	1	0,04	0,93-1,07	1,75	0,92	0,11	0,71-1,12		2,57	1,35	0,15	1,05-1,65	*
60-69 years	2,65	1	0,04	0,93-1,07	2,33	0,88	0,10	0,68-1,07		1,89	0,71	0,13	0,45-0,98	**
70-79 years	3,29	1	0,04	0,92-1,08	2,27	0,69	0,10	0,49-0,89	**	3,28	1,00	0,23	0,55-1,44	
80 years and older	3,99	1	0,05	0,91-1,09	3,24	0,81	0,14	0,53-1,10		4,96	1,24	0,47	0,32-2,17	
Total	1,46	1	0,02	0,97-1,03	1,44	0,99	0,05	0,90-1,09		2,70	1,86	0,07	1,71-2,00	*
**Suicide by drowning**														
<10 years	-	-	-	-	-	-	-	-		-	-	-	-	
10-19 years	0,02	1	0,41	0,20-1,80	0,07	3,78	2,68	-1,46-9,03		0,21	10,80	2,99	4,91-16,62	*
20-29 years	0,14	1	0,15	0,70-1,30	0,22	1,54	0,51	0,53-2,55		0,50	3,49	0,61	2,30-4,69	*
30-39 years	0,31	1	0,10	0,81-1,19	0,42	1,34	0,30	0,75-1,93		0,82	2,62	0,36	1,91-3,33	*
40-49 years	0,53	1	0,07	0,87-1,13	0,42	0,79	0,18	0,44-1,14		0,85	1,59	0,25	1,11-2,07	*
50-59 years	1,03	1	0,05	0,90-1,10	0,92	0,89	0,14	0,61-1,17		1,28	1,24	0,20	0,85-1,64	
60-69 years	1,47	1	0,05	0,91-1,09	1,28	0,87	0,13	0,61-1,13		1,28	0,87	0,20	0,48-1,26	
70-79 years	1,91	1	0,05	0,90-1,10	1,37	0,72	0,13	0,46-0,98	**	1,38	0,72	0,26	0,22-1,22	
80 years and older	2,09	1	0,06	0,87-1,13	2,13	1,02	0,22	0,58-1,45		2,84	1,36	0,68	0,03-2,69	
Total	0,68	1	0,02	0,95-1,05	0,62	0,91	0,07	0,78-1,04		1,16	1,69	0,12	1,46-1,92	*
**Accidental drowning**														
<10 years	0,59	1	0,08	0,85-1,15	0,60	1,03	0,26	0,52-1,53		1,56	2,60	0,27	2,07-3,13	*
10-19 years	0,14	1	0,15	0,70-1,30	0,44	3,17	0,91	1,38-4,96	*	0,71	5,09	0,77	3,58-6,59	*
20-29 years	0,16	1	0,14	0,72-1,28	0,32	1,96	0,54	0,89-3,02		0,64	3,91	0,60	2,73-5,10	*
30-39 years	0,20	1	0,12	0,77-1,23	0,59	2,87	0,54	1,81-3,93	*	0,58	2,85	0,47	1,93-3,76	*
40-49 years	0,37	1	0,08	0,84-1,16	0,46	1,25	0,27	0,73-1,77		0,61	1,63	0,30	1,04-2,21	*
50-59 years	0,55	1	0,07	0,86-1,14	0,67	1,20	0,22	0,77-1,64		1,01	1,83	0,33	1,17-2,48	*
60-69 years	0,80	1	0,07	0,87-1,13	0,70	0,87	0,18	0,52-1,22		0,40	0,51	0,21	0,10-0,91	**
70-79 years	0,92	1	0,07	0,85-1,15	0,57	0,62	0,18	0,27-0,97	**	1,21	1,32	0,50	0,34-2,30	
80 years and older	1,31	1	0,08	0,86-1,16	0,71	0,54	0,20	0,14-0,94	**	2,13	1,62	0,93	-0,21-3,45	
Total	0,48	1	0,03	0,94-1,06	0,57	1,19	0,09	1,01-1,37	*	1,15	2,42	0,14	2,14-2,69	*
**Transport accidents with drowning**														
<10 years	0,04	1	0,30	0,41-1,59	0,08	2,02	1,43	-0,78-4,82		0,11	1,47	1,12	-0,73-3,67	
10-19 years	0,14	1	0,15	0,70-1,30	0,19	1,32	0,59	0,16-2,48		0,13	0,92	0,33	0,28-1,57	
20-29 years	0,42	1	0,09	0,83-1,17	0,30	0,71	0,20	0,31-1,11		0,34	0,80	0,17	0,47-1,14	
30-39 years	0,20	1	0,12	0,77-1,23	0,17	0,82	0,29	0,25-1,39		0,39	1,92	0,38	1,17-2,68	*
40-49 years	0,19	1	0,11	0,78-1,22	0,21	1,10	0,35	0,42-1,79		0,26	1,37	0,38	0,63-2,12	
50-59 years	0,26	1	0,10	0,80-1,20	0,09	0,36	0,18	0,01-0,71	**	0,24	0,92	0,35	0,24-1,61	
60-69 years	0,30	1	0,11	0,79-1,21	0,32	1,06	0,32	0,44-1,69		0,20	0,67	0,39	-0,09-1,43	
70-79 years	0,39	1	0,11	0,78-1,22	0,19	0,49	0,24	0,01-0,97	**	0,35	0,89	0,63	-0,34-2,13	
80 years and older	0,50	1	0,13	0,74-1,26	0,41	0,81	0,40	0,02-1,60		-	-	-	-	
Total	0,25	1	0,04	0,92-1,08	0,20	0,80	0,10	0,60-1,00		0,28	1,15	0,12	0,91-1,39	

SM Standardized Mortality
SMR Standardized Mortality Ratio (mortality of residents with a Dutch background = 1)
SD Standard deviation
CI Confidence interval of 95%
sign. * = significant higher than residents with a Dutch background
** = significant lower than residents with a Dutch background
