# Supplementary material for: Fatal drowning statistics from the Netherlands – an example of an aggregated demographic profile
Source: BMC Public Health. 2022 Feb 17;22:339. doi: 10.1186/s12889-022-12620-3 (PMC8851711; doi:10.1186/s12889-022-12620-3)
Supplement: Supplementary file 3 — Additional file 3. Supplementary Table to Figure 3. Trend of fatal drowning in the Netherlands 1998–2017; by cause of drowning: trend of total number and of incidence per 100,000 of the population by year and cause of drowning. [file 12889_2022_12620_MOESM3_ESM.pdf]

Supplementary Table to Figure 3. Trend of fatal drowning in the Netherlands 1998-2017; by cause of drowning: trend of total number and of incidence per 100,000 of the population by year and cause of drowning

Supplementary Table to Figure 3a. Fatal drowning in the Netherlands 1998-2017; trend of total number by year and cause of drowning

	Suicide			Accidental drowning			Transport accidents with drowning			Residual drowning			Total		
	Males	Females	Total	Males	Females	Total	Males	Females	Total	Males	Females	Total	Males	Females	Total
	Number of fatal drowning														
1998	78	60	138	88	13	101	58	12	70	17	4	21	241	89	330
1999	73	53	126	84	23	107	41	8	49	14	2	16	212	86	298
2000	81	68	149	84	35	119	25	10	35	4	2	6	194	115	309
2001	79	45	124	87	19	106	23	14	37	9	5	14	198	83	281
2002	71	55	126	111	30	141	46	9	55	8	1	9	236	95	331
2003	64	43	107	82	26	108	39	7	46	13	4	17	198	80	278
2004	67	53	120	88	31	119	33	9	42	9	1	10	197	94	291
2005	69	47	116	92	19	111	35	9	44	6	0	6	202	75	277
2006	57	49	106	86	21	107	30	7	37	7	3	10	180	80	260
2007	52	38	90	77	17	94	27	11	38	8	1	9	164	67	231
2008	62	41	103	77	22	99	40	6	46	6	4	10	185	73	258
2009	58	53	111	88	25	113	33	14	47	5	0	5	184	92	276
2010	66	41	107	74	16	90	43	10	53	5	3	8	188	70	258
2011	69	39	108	78	15	93	29	9	38	6	3	9	182	66	248
2012	77	53	130	73	18	91	26	12	38	4	1	5	180	84	264
2013	66	44	110	73	27	100	31	9	40	4	0	4	174	80	254
2014	78	48	126	67	27	94	38	7	45	4	3	7	187	85	272
2015	73	49	122	82	20	102	36	5	41	5	1	6	196	75	271
2016	71	50	121	90	25	115	48	10	58	3	3	6	212	88	300
2017	75	51	126	88	19	107	37	9	46	4	1	5	204	80	284
1998-2007	691	511	1202	879	234	1113	357	96	453	95	23	118	2022	864	2886
2008-2017	753	552	1164	956	256	1004	397	102	452	101	27	65	2207	937	2685
1998-2017	1386	980	2366	1669	448	2117	718	187	905	141	42	183	3914	1657	5571

Supplementary Table to Figure 3b. Fatal drowning in the Netherlands 1998-2017; trend of incidence per 100,000 of the population by year and cause of drowning

	Suicide			Accidental drowning			Transport accidents with drowning			Residual drowning			Total		
	Males	Females	Total	Males	Females	Total	Males	Females	Total	Males	Females	Total	Males	Females	Total
	per 100 000 of the population														
1998	1,00	0,76	0,88	1,13	0,16	0,64	0,75	0,15	0,45	0,22	0,05	0,13	3,10	1,12	2,10
1999	0,93	0,66	0,80	1,07	0,29	0,68	0,52	0,10	0,31	0,18	0,03	0,10	2,71	1,08	1,88
2000	1,03	0,84	0,94	1,07	0,43	0,75	0,32	0,12	0,22	0,05	0,02	0,04	2,46	1,43	1,94
2001	0,99	0,56	0,77	1,10	0,23	0,66	0,29	0,17	0,23	0,11	0,06	0,09	2,49	1,02	1,75
2002	0,89	0,67	0,78	1,39	0,37	0,87	0,58	0,11	0,34	0,10	0,01	0,06	2,95	1,16	2,05
2003	0,80	0,52	0,66	1,02	0,32	0,67	0,49	0,09	0,28	0,16	0,05	0,10	2,47	0,98	1,71
2004	0,83	0,64	0,74	1,09	0,38	0,73	0,41	0,11	0,26	0,11	0,01	0,06	2,45	1,14	1,79
2005	0,85	0,57	0,71	1,14	0,23	0,68	0,43	0,11	0,27	0,07	0,00	0,04	2,50	0,91	1,70
2006	0,71	0,59	0,65	1,06	0,25	0,65	0,37	0,08	0,23	0,09	0,04	0,06	2,23	0,97	1,59
2007	0,64	0,46	0,55	0,95	0,21	0,57	0,33	0,13	0,23	0,10	0,01	0,05	2,02	0,81	1,41
2008	0,76	0,49	0,63	0,95	0,26	0,60	0,49	0,07	0,28	0,07	0,05	0,06	2,27	0,88	1,57
2009	0,71	0,63	0,67	1,08	0,30	0,68	0,40	0,17	0,28	0,06	0,00	0,03	2,25	1,10	1,67
2010	0,80	0,49	0,64	0,90	0,19	0,54	0,52	0,12	0,32	0,06	0,04	0,05	2,29	0,83	1,55
2011	0,84	0,46	0,65	0,94	0,18	0,56	0,35	0,11	0,23	0,07	0,04	0,05	2,20	0,78	1,49
2012	0,93	0,63	0,78	0,88	0,21	0,54	0,31	0,14	0,23	0,05	0,01	0,03	2,17	0,99	1,58
2013	0,79	0,52	0,65	0,88	0,32	0,60	0,37	0,11	0,24	0,05	0,00	0,02	2,09	0,94	1,51
2014	0,93	0,56	0,75	0,80	0,32	0,56	0,45	0,08	0,27	0,05	0,04	0,04	2,24	1,00	1,61
2015	0,87	0,57	0,72	0,98	0,23	0,60	0,43	0,06	0,24	0,06	0,01	0,04	2,33	0,88	1,60
2016	0,84	0,58	0,71	1,07	0,29	0,68	0,57	0,12	0,34	0,04	0,03	0,04	2,51	1,03	1,76
2017	0,88	0,59	0,74	1,04	0,22	0,62	0,44	0,10	0,27	0,05	0,01	0,03	2,40	0,93	1,66
1998-2007	0,87	0,63	0,75	1,10	0,29	0,69	0,45	0,12	0,28	0,12	0,03	0,07	2,54	1,06	1,79
2008-2017	0,91	0,65	0,69	1,15	0,30	0,60	0,48	0,12	0,27	0,12	0,03	0,04	2,66	1,11	1,60
1998-2017	0,85	0,59	0,72	1,02	0,27	0,64	0,44	0,11	0,28	0,09	0,03	0,06	2,40	1,00	1,69
