# Supplementary material for: Fatal drowning statistics from the Netherlands – an example of an aggregated demographic profile
Source: BMC Public Health. 2022 Feb 17;22:339. doi: 10.1186/s12889-022-12620-3 (PMC8851711; doi:10.1186/s12889-022-12620-3)
Supplement: Supplementary file 4 — Additional file 4. Supplementary Table to Figure 4. Trends of fatal drowning in the Netherlands 1998–2017; incidence per 100,000 of the population by cause of drowning; single regression analysis and breakpoint analysis. Additional information of the breakpoint analysis. [file 12889_2022_12620_MOESM4_ESM.pdf]

Supplementary Table to Figure 4. Trends of fatal drowning in the Netherlands 1998-2017; incidence per 100,000 of the population by cause of drowning; single regression analysis and breakpoint analysis. Additional information of the breakpoint analysis.

Total drowning

Groep x-a	X-as label	X-axis EN	Total Dro	Trend_1	Trend_2	General T
	1998		2,10	2,06		1,89
	1999		1,88	2,00		1,87
	2000		1,94	1,94		1,85
	2001		1,75	1,88		1,83
	2002		2,05	1,82		1,81
	2003		1,71	1,76		1,79
	2004		1,79	1,70		1,77
	2005		1,70	1,65		1,75
	2006		1,59	1,59		1,73
	2007		1,41		1,53	1,71
	2008		1,57		1,55	1,69
	2009		1,67		1,56	1,67
	2010		1,55		1,57	1,65
	2011		1,49		1,58	1,62
	2012		1,58		1,59	1,60
	2013		1,51		1,61	1,58
	2014		1,61		1,62	1,56
	2015		1,60		1,63	1,54
	2016		1,76		1,64	1,52
	2017		1,66		1,65	1,50

Suicide by drowning

Groep x-a	X-as label	X-axis EN	Suicide by	Trend_1	Trend_2	General T
	1998		0,88	0,89		0,78
	1999		0,80	0,86		0,78
	2000		0,94	0,83		0,77
	2001		0,77	0,80		0,76
	2002		0,78	0,76		0,76
	2003		0,66	0,73		0,75
	2004		0,74	0,70		0,74
	2005		0,71	0,67		0,74
	2006		0,65	0,63		0,73
	2007		0,55	0,60		0,72
	2008		0,63		0,64	0,72
	2009		0,67		0,65	0,71
	2010		0,64		0,67	0,70
	2011		0,65		0,68	0,70
	2012		0,78		0,69	0,69
	2013		0,65		0,70	0,68
	2014		0,75		0,71	0,68
	2015		0,72		0,72	0,67
	2016		0,71		0,73	0,66
	2017		0,74		0,74	0,66

Accidental drowning

Groep x-as	X-as label	X-axis EN	Accidental	Trend_1	Trend_2	Trend_3	General Tr
	1998		0,64	0,66			0,72
	1999		0,68	0,68			0,71
	2000		0,75	0,69			0,70
	2001		0,67	0,70			0,69
	2002		0,87		0,77		0,69
	2003		0,67		0,75		0,68
	2004		0,73		0,72		0,67
	2005		0,68		0,70		0,66
	2006		0,65		0,67		0,66
	2007		0,58		0,65		0,65
	2008		0,60		0,62		0,64
	2009		0,68		0,59		0,63
	2010		0,55		0,57		0,63
	2011		0,56		0,54		0,62
	2012		0,54			0,55	0,61
	2013		0,60			0,57	0,61
	2014		0,56			0,59	0,60
	2015		0,60			0,61	0,59
	2016		0,68			0,63	0,58
	2017		0,63			0,65	0,58

Transport accidents with drowning

Groep x-as	X-as label	X-axis EN	transporta	Trend_1
	1998		0,45	0,30
	1999		0,31	0,30
	2000		0,22	0,30
	2001		0,23	0,29
	2002		0,34	0,29
	2003		0,28	0,29
	2004		0,26	0,28
	2005		0,27	0,28
	2006		0,23	0,28
	2007		0,23	0,28
	2008		0,28	0,27
	2009		0,28	0,27
	2010		0,32	0,27
	2011		0,23	0,27
	2012		0,23	0,26
	2013		0,24	0,26
	2014		0,27	0,26
	2015		0,24	0,26
	2016		0,34	0,25
	2017		0,26	0,25
