# Supplementary material for: Fatal drowning statistics from the Netherlands – an example of an aggregated demographic profile
Source: BMC Public Health. 2022 Feb 17;22:339. doi: 10.1186/s12889-022-12620-3 (PMC8851711; doi:10.1186/s12889-022-12620-3)
Supplement: Supplementary file 5 — Additional file 5. Supplementary Table 3. Fatal drowning in the Netherlands 1998–2017; comparison between 1998 and 2007 and 2008–2017 by cause of drowning and age group. The mortality ratio of 2008–2017 is age-adjusted (SM) based on the population 1998–2007. Additional information on Standard Mortality, Standard Mortality Ratio, Deviation Rate and 95% Confidence Interval. [file 12889_2022_12620_MOESM5_ESM.pdf]

Supplementary Table 3. Fatal drowning in the Netherlands 1998-2017; comparison between 1998-2007 and 2008-2017 by cause of drowning and age group. The mortality ratio of 2008-2017 is age-adjusted (SM) based on the population 1998-2007. Additional information on Standard Mortality, Standard Mortality Ratio, Deviation Rate and 95% Confidence Interval. For age-specific data incidence and SM are identical.

Supplementary Table 3
Fatal drownings in the Netherlands 1998-2017, comparison between 1998-2007 and 2008-2017; per 100,000 of the population by cause of drowning and age group

	1998-2007				2008-2017				
	SM	SMR	SD	CI (95%)	SM	SMR	SD	CI (95%)	sign.
**Total drowning**									
<10 years	1,20	1	0,06	0,87-1,13	0,58	0,49	0,05	0,39-0,58	**
10-19 years	0,61	1	0,09	0,82-1,18	0,52	0,85	0,08	0,69-1,02	
20-29 years	1,33	1	0,06	0,88-1,12	1,18	0,89	0,06	0,78-1,01	
30-39 years	1,23	1	0,06	0,89-1,11	1,25	1,02	0,06	0,90-1,14	
40-49 years	1,62	1	0,05	0,90-1,10	1,35	0,83	0,05	0,74-0,92	**
50-59 years	2,38	1	0,04	0,91-1,09	1,97	0,82	0,04	0,75-0,90	**
60-69 years	3,14	1	0,05	0,91-1,09	2,49	0,79	0,04	0,72-0,86	**
70-79 years	3,40	1	0,05	0,90-1,10	3,33	0,98	0,05	0,88-1,08	
80 years and older	4,28	1	0,07	0,87-1,13	3,84	0,90	0,05	0,79-1,00	
Total	1,79	1	0,02	0,96-1,04	1,51	0,85	0,02	0,81-0,88	**
**Suicide by drowning**									
<10 years	-	-	-	-	-	-	-	-	
10-19 years	0,07	1	0,27	0,48-1,52	0,06	0,83	0,24	0,36-1,30	
20-29 years	0,32	1	0,12	0,76-1,24	0,28	0,86	0,11	0,64-1,09	
30-39 years	0,45	1	0,09	0,82-1,18	0,47	1,04	0,11	0,84-1,25	
40-49 years	0,69	1	0,08	0,85-1,15	0,53	0,77	0,07	0,64-0,90	**
50-59 years	1,24	1	0,06	0,88-1,12	1,00	0,81	0,05	0,70-0,91	**
60-69 years	1,65	1	0,06	0,87-1,13	1,36	0,83	0,05	0,73-0,93	**
70-79 years	1,98	1	0,07	0,86-1,14	1,85	0,94	0,06	0,81-1,06	
80 years and older	2,42	1	0,09	0,83-1,17	1,93	0,80	0,07	0,66-0,93	**
Total	0,75	1	0,03	0,94-1,06	0,63	0,85	0,02	0,80-0,90	**
**Accidental drowning**									
<10 years	1,11	1	0,07	0,87-1,13	0,51	0,46	0,05	0,37-0,55	**
10-19 years	0,33	1	0,13	0,76-1,24	0,32	0,98	0,12	0,74-1,22	
20-29 years	0,37	1	0,11	0,78-1,22	0,52	1,40	0,13	1,13-1,66	*
30-39 years	0,45	1	0,09	0,82-1,18	0,48	1,08	0,11	0,87-1,29	
40-49 years	0,60	1	0,08	0,84-1,16	0,51	0,85	0,08	0,71-1,00	
50-59 years	0,79	1	0,08	0,85-1,15	0,65	0,80	0,07	0,67-0,92	**
60-69 years	1,04	1	0,08	0,84-1,16	0,76	0,73	0,06	0,62-0,85	**
70-79 years	1,01	1	0,10	0,81-1,19	0,94	0,93	0,09	0,76-1,10	
80 years and older	1,26	1	0,12	0,76-1,24	1,37	1,09	0,11	0,87-1,30	
Total	0,69	1	0,03	0,94-1,06	0,58	0,84	0,03	0,79-0,89	**
**Transport accidents with drowning**									
<10 years	0,06	1	0,30	0,41-1,59	0,05	0,97	0,31	0,37-1,57	
10-19 years	0,19	1	0,16	0,68-1,32	0,13	0,71	0,14	0,44-0,97	**
20-29 years	0,55	1	0,09	0,81-1,19	0,34	0,62	0,07	0,48-0,77	**
30-39 years	0,26	1	0,12	0,76-1,24	0,27	1,01	0,14	0,75-1,28	
40-49 years	0,24	1	0,13	0,74-1,26	0,27	1,12	0,14	0,86-1,39	
50-59 years	0,29	1	0,13	0,75-1,25	0,28	0,95	0,12	0,72-1,18	
60-69 years	0,33	1	0,14	0,72-1,28	0,33	1,01	0,12	0,76-1,25	
70-79 years	0,31	1	0,18	0,65-1,35	0,45	1,46	0,20	1,07-1,84	*
80 years and older	0,48	1	0,20	0,62-1,38	0,51	1,08	0,18	0,73-1,43	
Total	0,28	1	0,05	0,91-1,09	0,26	0,94	0,04	0,85-1,03	

SM	Standardized Mortality
SMR	Standardized Mortality Ratio (mortality in 1998-2007 = 1)
SD	Standard deviation
CI	Confidence interval of 95%
sign.	* = significant higher than in the period 1998-2007
	** = significant lower than in the period 1998-2007
