# Supplementary material for: Fatal drowning statistics from the Netherlands – an example of an aggregated demographic profile
Source: BMC Public Health. 2022 Feb 17;22:339. doi: 10.1186/s12889-022-12620-3 (PMC8851711; doi:10.1186/s12889-022-12620-3)
Supplement: Supplementary file 6 — Additional file 6. Supplementary Table 4. Fatal drowning in the Netherlands 1998–2017; comparison between 1998 and 2007 and 2008–2017; Additional information on Standard Mortality, Standard Mortality Ratio, Deviation Rate and 95% Confidence Interval by cause of drowning and migration background. [file 12889_2022_12620_MOESM6_ESM.pdf]

Supplementary Table 4. Fatal drowning in the Netherlands 1998-2017; comparison between 1998-2007 and 2008-2017; Additional information on Standard Mortality, Standard Mortality Ratio, Deviation Rate and 95% Confidence Interval by cause of drowning and migration background. For age-specific data incidence and SM are identical.

Supplementary Table 4

Fatal drownings in the Netherlands 1998-2017, comparison between 1998-2007 and 2008-2017; per 100,000 of the population by migration background, cause of drowning and age group

	Dutch background					Western background					Non-western background				
	1998-2007	2008-2017				1998-2007	2008-2017				1998-2007	2008-2017			
	SM	SM	SMR	CI (95%)	sign.	SM	SM	SMR	CI (95%)	sign.	SM	SM	SMR	CI (95%)	sign.
**Total drowning**															
<10 years	0,84	0,44	0,52	0,39-0,66	**	0,96	0,43	0,45	0,09-0,80	**	2,38	1,07	0,45	0,30-0,60	**
10-19 years	0,36	0,27	0,73	0,51-0,96	**	0,45	0,95	2,09	0,96-3,23		1,24	0,92	0,74	0,48-1,10	
20-29 years	0,92	0,62	0,68	0,54-0,82	**	1,25	0,59	0,47	0,21-0,72	**	1,58	1,67	1,05	0,79-1,32	
30-39 years	0,79	0,71	0,90	0,73-1,07		1,04	1,47	1,41	0,94-1,88		2,19	1,70	0,78	0,58-0,98	**
40-49 years	1,34	0,96	0,71	0,61-0,82	**	1,41	1,05	0,75	0,46-1,04		2,24	1,53	0,68	0,48-0,88	**
50-59 years	2,12	1,70	0,80	0,72-0,89	**	2,20	1,31	0,60	0,38-0,81	**	2,29	2,72	1,19	0,87-1,51	
60-69 years	3,03	2,37	0,78	0,70-0,86	**	2,24	2,39	1,07	0,76-1,37		2,07	1,79	0,86	0,45-1,27	
70-79 years	3,31	3,27	0,99	0,88-1,09		2,48	2,12	0,85	0,53-1,18		3,78	3,09	0,82	0,37-1,26	
80 years and older	4,28	3,76	0,88	0,77-0,99	**	2,27	3,90	1,72	1,02-2,42	*	9,97	2,97	0,30	-0,04-0,64	**
Total	1,55	1,25	0,81	0,77-0,84	**	1,50	1,34	0,89	0,78-1,01		1,99	1,54	0,77	0,69-0,86	**
**Suicide by drowning**															
<10 years	-	-	-	-		-	-	-	-		-	-	-	-	
10-19 years	0,02	0,02	0,99	-0,13-2,10		0,00	-	-	-		0,31	0,12	0,39	0,01-0,78	**
20-29 years	0,16	0,13	0,79	0,44-1,15		0,38	0,09	0,24	-0,09-0,57	**	0,44	0,56	1,27	0,71-1,83	
30-39 years	0,32	0,30	0,92	0,65-1,18		0,38	0,46	1,23	0,50-1,96		0,84	0,79	0,94	0,58-1,29	
40-49 years	0,61	0,45	0,74	0,59-0,90	**	0,53	0,32	0,61	0,19-1,04		1,09	0,68	0,62	0,35-0,89	**
50-59 years	1,16	0,92	0,79	0,67-0,90	**	1,12	0,72	0,65	0,33-0,96	**	0,95	1,47	1,54	0,97-2,11	
60-69 years	1,66	1,32	0,80	0,69-0,90	**	1,12	1,40	1,25	0,78-1,72		1,32	1,26	0,96	0,42-1,50	
70-79 years	1,91	1,92	1,00	0,86-1,14		1,69	1,14	0,67	0,32-1,03		3,15	0,71	0,23	-0,03-0,48	**
80 years and older	2,39	1,86	0,78	0,64-0,92	**	1,77	2,37	1,34	0,64-2,05		7,48	0,99	0,13	-0,13-0,39	**
Total	0,71	0,59	0,83	0,78-0,89	**	0,65	0,56	0,87	0,69-1,05		0,60	0,50	0,83	0,68-0,98	**
**Accidental drowning**															
<10 years	0,76	0,39	0,51	0,38-0,65	**	0,96	0,28	0,30	0,01-0,59	**	2,22	0,91	0,41	0,26-0,56	**
10-19 years	0,16	0,12	0,78	0,43-1,13		0,30	0,58	1,93	0,59-3,27		0,69	0,74	1,07	0,64-1,19	
20-29 years	0,16	0,17	1,04	0,64-1,45		0,27	0,36	1,33	0,41-2,25		0,61	0,67	1,10	0,66-1,54	
30-39 years	0,22	0,18	0,84	0,53-1,15		0,38	0,80	2,13	1,17-3,08	*	0,74	0,44	0,59	0,29-0,89	**
40-49 years	0,45	0,29	0,64	0,48-0,81	**	0,48	0,45	0,92	0,38-1,46		0,65	0,58	0,89	0,47-1,32	
50-59 years	0,64	0,47	0,74	0,59-0,89	**	0,84	0,50	0,59	0,24-0,94	**	0,95	1,05	1,10	0,62-1,58	
60-69 years	0,91	0,72	0,79	0,65-0,93	**	0,92	0,52	0,56	0,21-0,91	**	0,38	0,42	1,12	0,02-2,21	
70-79 years	0,97	0,87	0,90	0,72-1,09		0,56	0,57	1,01	0,26-1,76		0,63	1,43	2,26	0,45-4,07	
80 years and older	1,23	1,38	1,12	0,88-1,35		0,50	0,85	1,68	0,21-3,15		2,49	1,98	0,79	-0,31-1,90	
Total	0,52	0,41	0,78	0,72-0,85	**	0,57	0,52	0,91	0,72-1,11		1,00	0,75	0,76	0,63-0,88	**
**Transport accidents with drowning**															
<10 years	0,05	0,03	0,63	0,01-1,25		0,00	-	-	-		0,10	0,13	1,32	0,03-2,61	
10-19 years	0,16	0,12	0,71	0,38-1,04		0,15	0,22	1,45	-0,19-3,09		0,21	0,06	0,30	-0,11-0,71	**
20-29 years	0,53	0,30	0,57	0,40-0,73	**	0,49	0,14	0,28	-0,04-0,59	**	0,37	0,31	0,83	0,34-1,31	
30-39 years	0,21	0,20	0,95	0,61-1,28		0,17	0,17	1,01	0,02-2,00		0,37	0,41	1,11	0,53-1,69	
40-49 years	0,20	0,18	0,92	0,62-1,22		0,22	0,20	0,92	0,11-1,73		0,35	0,20	0,58	0,12-1,05	
50-59 years	0,26	0,25	0,97	0,70-1,24		0,14	0,05	0,32	-0,31-0,96	**	0,38	0,16	0,41	-0,05-0,88	**
60-69 years	0,31	0,29	0,94	0,68-1,20		0,20	0,42	2,10	0,65-3,56		0,38	0,11	0,28	-0,27-0,83	**
70-79 years	0,35	0,42	1,21	0,85-1,57		-	-	-	-		-	-	-	-	
80 years and older	0,52	0,49	0,95	0,62-1,28		-	-	-	-		-	-	-	-	
Total	0,26	0,22	0,87	0,77-0,96	**	0,19	0,22	1,16	0,77-1,55		0,28	0,21	0,76	0,53-0,98	**

SM	Standardized Mortality
SMR	Standardized Mortality Ratio (mortality in the period 1998-2007 = 1)
CI	Confidence interval of 95%
sign.	* = significant higher than in the period 1998-2007
	** = significant lower than in the period 1998-2007
